# Novel Split Chest Tube Improves Post-Surgical Thoracic Drainage

**DOI:** 10.4172/2155-9880.1000321

**Published:** 2014-06-30

**Authors:** Albert H Olivencia-Yurvati, Brandon H Cherry, Hunaid A Gurji, Daniel W White, J Tyler Newton, Gary F Scott, Besim Hoxha, Terence Gourlay, Robert T Mallet

**Affiliations:** 1 Departments of Surgery, University of North Texas Health Science Center, Fort Worth, TX, USA; 2 Departments of Integrative Physiology, University of North Texas Health Science Center, Fort Worth, TX, USA; 3 Cardiovascular Research Institute, University of North Texas Health Science Center, Fort Worth, TX, USA; 4 Bioengineering Unit, Wolfson Centre, University of Strathclyde, Glasgow, UK

**Keywords:** Chest, Pleural effusion, Pleural space, Surgical equipment, Thoracotomy

## Abstract

**Objective:**

Conventional, separate mediastinal and pleural tubes are often inefficient at draining thoracic effusions.

**Description:**

We developed a Y-shaped chest tube with split ends that divide within the thoracic cavity, permitting separate intrathoracic placement and requiring a single exit port. In this study, thoracic drainage by the split drain vs. that of separate drains was tested.

**Methods:**

After sternotomy, pericardiotomy, and left pleurotomy, pigs were fitted with separate chest drains (n=10) or a split tube prototype (n=9) with internal openings positioned in the mediastinum and in the costo-diaphragmatic recess. Separate series of experiments were conducted to test drainage of D5W or 0.58 M sucrose, an aqueous solution with viscosity approximating that of plasma. One litre of fluid was infused into the thorax, and suction was applied at −20 cm H2O for 30 min.

**Results:**

When D5W was infused, the split drain left a residual volume of 53 ± 99 ml (mean value ± SD) vs. 148 ± 120 for the separate drain (P=0.007), representing a drainage efficiency (i.e. drained vol/[drained + residual vol]) of 95 ± 10% vs. 86 ± 12% for the separate drains (P = 0.011). In the second series, the split drain evacuated more 0.58 M sucrose in the first minute (967 ± 129 ml) than the separate drains (680 ± 192 ml, P<0.001). By 30 min, the split drain evacuated a similar volume of sucrose vs. the conventional drain (1089 ± 72 vs. 1056 ± 78 ml; P = 0.5). Residual volume tended to be lower (25 ± 10 vs. 62 ± 72 ml; P = 0.128) and drainage efficiency tended to be higher (98 ± 1 vs. 95 ± 6%; P = 0.111) with the split drain vs. conventional separate drains.

**Conclusion:**

The split chest tube drained the thoracic cavity at least as effectively as conventional separate tubes. This new device could potentially alleviate postoperative complications.

## Introduction

Lifesaving surgeries on the heart and lungs, including coronary artery bypass grafting, replacement of diseased heart valves, and resection of lung cancers, require opening the chest cavity, incising the pericardial and/or pleural membranes lining the chest wall and thoracic organs, and operating on the heart, lungs and/or major blood vessels. These surgical sites can exude large volumes of fluid, often exceeding 1 litre, which collects in the pleural space and compresses the thoracic organs, compromising cardiac performance and ventilation [1-9]. Indeed, residual pleural effusions and drain discomfort persist as significant post-operative morbidities [2,10]. Although data on the true incidence of residual effusions is limited, clinically significant post-operative pleural effusions occur in 10-40% of patients recovering from open heart surgery [2,9].

To drain these effusions and relieve congestion of the thoracic cavity, tubes are placed in the mediastinal and pleural spaces. Conventional drainage systems require two insertion sites, which are more painful and impose a greater risk of infection than a single insertion site. Moreover, these tubes often fail to effectively drain the chest cavity. Large volumes of pleural fluid can collect in the costo-diaphragmatic recess within the left pleural space. To effectively drain this fluid, the tubing must be tightly curved to direct it caudally and posteriorly into the recess. Because conventional tubing lacks embedded structures to hold its curvature, it can slip out of position, interrupting pleural drainage. However, no new devices have been developed to address this clinical problem. Improved chest drain device and performance can reduce drain indwelling duration so drains can be removed earlier, allowing hospitalization to be shortened from the current 4-5 days [11-13].

The limitations of the separate chest drains prompted development of a new configuration which allows the placement of a single drain that, once inserted into the thoracic cavity, affords the option to split into a dual drain. Experiments were conducted in pigs to evaluate and compare the rate and efficiency of fluid drainage by the split tubing system vs. conventional, separate tubes. The new system evacuated fluid at least as effectively as the separate tubes, and did so more rapidly, leaving less residual fluid within the thoracic recesses.

## Methods

### Split chest drain prototype

**Figure 1:**
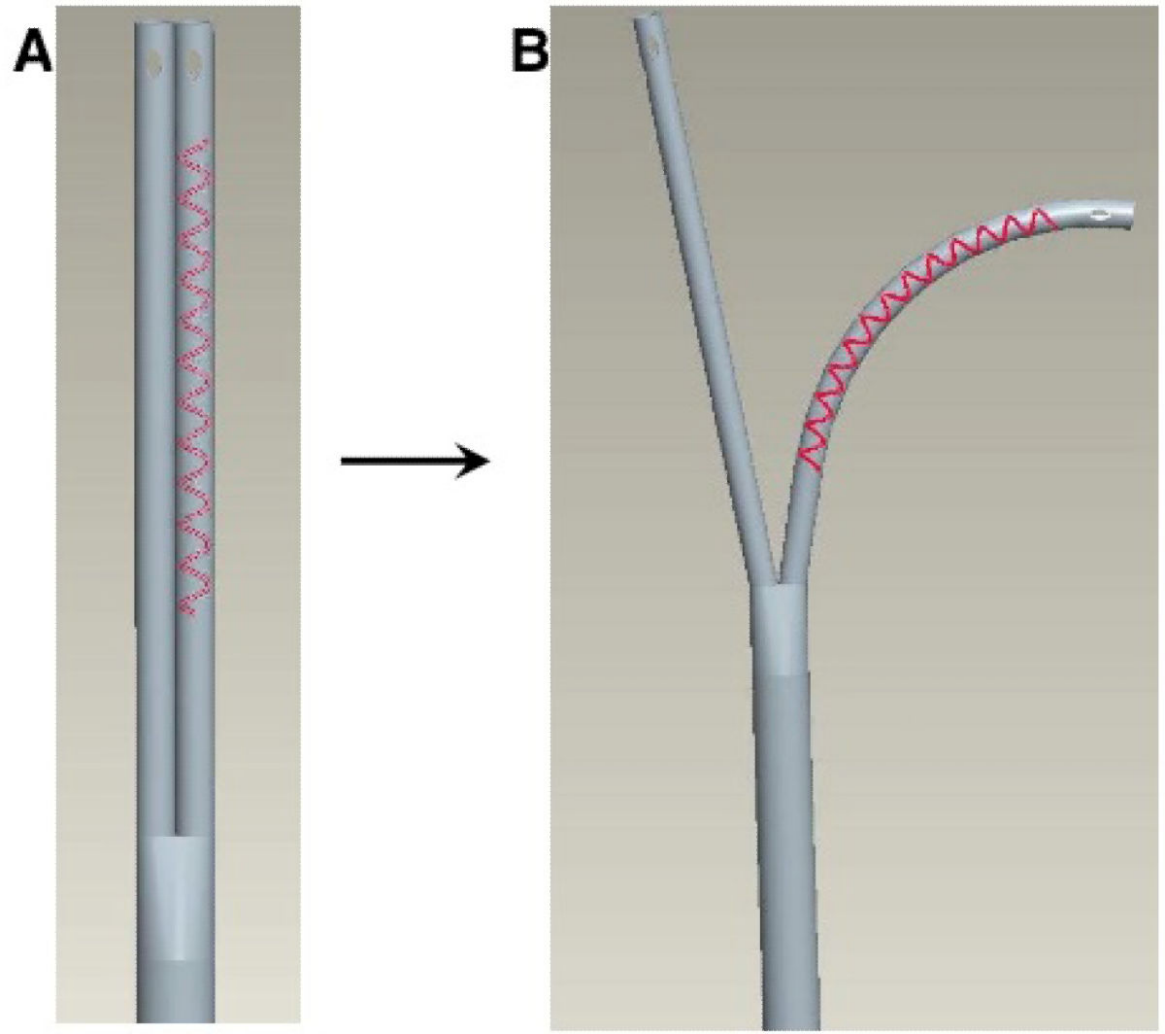
Split chest drain prototype. Within the chest, the two arms are divided and placed in separate locations. These tubes empty into a common drain which is exteriorized through the chest wall. A malleable external wire is coiled around the pleural arm to provide deformability and hold the placement and curvature of the tubing within the costo-diaphragmatic recess. Future refinements of this device will utilize a wire embedded within the wall of the tubing.

A prototype of the split chest drain design was fashioned from Atrium™ silicone thoracic catheters (24 F caliber). Wire (1 mm diameter) was tightly coiled around the exterior surface of one of the two intrathoracic arms to hold the curvature and position of this segment after its placement in the costo-diaphragmatic recess. The segment of the catheter with coiled wire was wrapped in a proprietary biocompatible film to prevent trauma and/or inflammation from the wire (Figure 1).

### Evaluation of Thoracic Drainage by Split Drain Prototype vs. Separate Drains

Experiments were conducted to evaluate and compare the thoracic drainage effectiveness of the split chest drain against that of a conventional system employing two separate tubes. All animal experimentation was approved by the Animal Care and Use Committee of the University of North Texas Health Science Centre, and conformed with the Guide to the Care and Use of Laboratory Animals [14]. Pigs (c. 50 kg) were sedated with Telazol/xylazine (5 mg/kg, im), and ventilated with 1-2% isoflurane to maintain anaesthesia.

**Figure 2:**
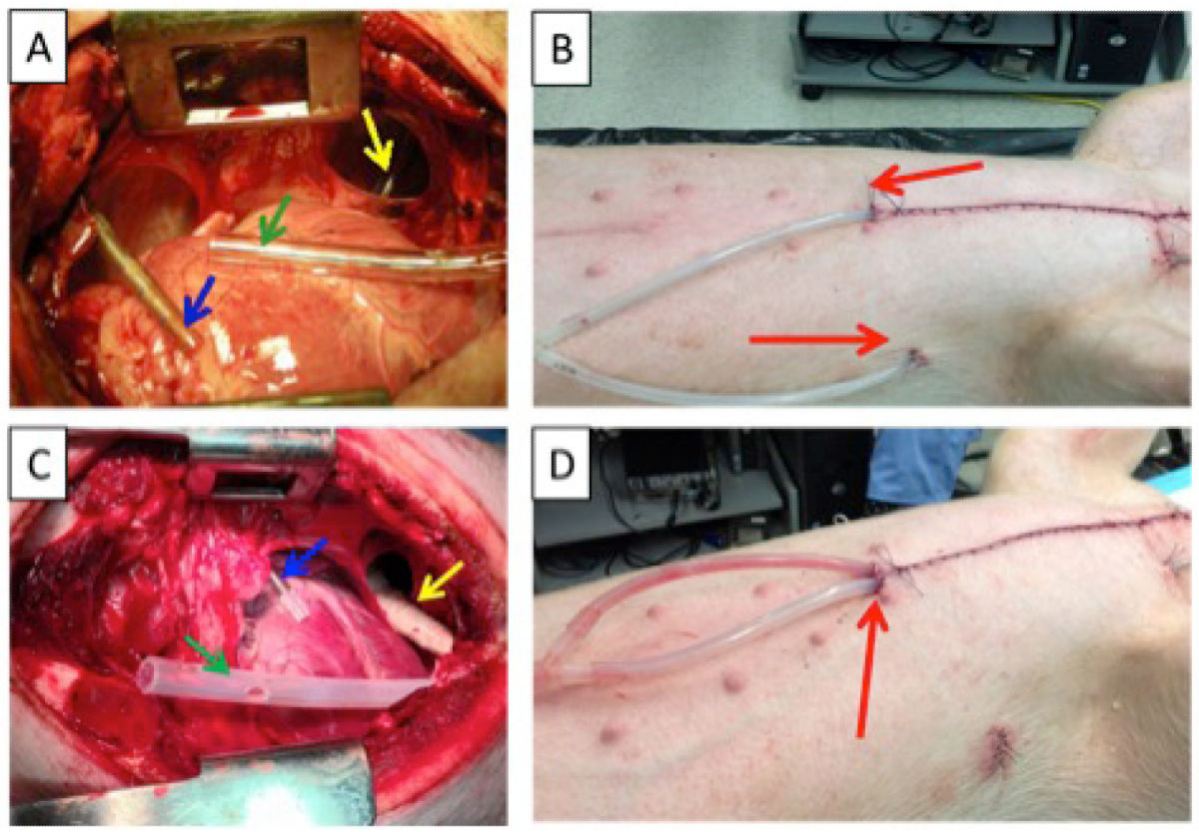
Thoracic placement of conventional drainage tubes (A, B) and prototype split chest drain (C, D). Panels A and C show placement of drainage tubes within the thoracic cavity. Blue arrows: catheter for sucrose infusion; green arrows: mediastinal drain; yellow arrows: pleural drain in the costo-diaphragmatic recess. Panels B and D show the exit incisions (red arrows) for the drainage tubes after closure of the thorax.

Each pig underwent a median sternotomy, pericardiotomy, and left pleurotomy. Next, chest tubes were inserted into the thoracic cavity. A 3 cm incision was made on the skin, a Schnidt clamp was used for blunt dissection and to secure and exteriorize the drainage catheter via the incision, and the catheter was secured at the incision with a purse string suture (Figure 2B, D: red arrows). In the conventional drainage configuration, two separate tubes were positioned in the mediastinum (Figure 2A: green arrow) and within the costo-diaphragmatic recess (Figure 2A: yellow arrow). In the split drain configuration, the tubing was divided, the intrathoracic ends positioned to drain the mediastinum (Figure 2C: green arrow) and costo-diaphragmatic recess (Figure 2C: yellow arrow), and the common end exteriorized through the parasternal angle.

In each pig an infusion catheter was placed in the upper thorax via the 2nd intercostal space to deliver 1 liter of fluid (Figure 2A, C: blue arrows). In the first series of experiments, an aqueous solution of 5% dextrose (D5W) was infused into 9 pigs with the separate drainage configuration and 12 pigs with the split drain. In the second series (n=10 separate drainage, 9 split drainage), an aqueous solution of 0.58 M sucrose was infused which approximates the viscosity of plasma (c. 1.8× the viscosity of pure water), the principal source of post-thoracic surgical effusions [15,16].

After surgical instrumentation was complete, the sternum was approximated with wires, and the thoracotomy sutured closed. 500 ml of fluid was infused into the thorax, a dry suction water seal chest drain was initiated at −20 cm H_2_O and an additional 500 ml was allowed to freely flow into the chest cavity, providing a total of 1000 ml of infused fluid. Extracted fluid was collected in a graduated chamber and its volume measured at 1, 5, 10, 20 and 30 min suction. After 30 min suction, the chest cavity was re-opened and the residual volume was extracted into a separate graduated container for measurement. Drainage efficiency was calculated as extracted fluid volume divided by the sum of extracted and residual fluid volumes.

### Statistical Analyses

Results are reported as mean values ± SD. Cumulative drainage output values at the different time points were compared by a two-factor (drainage configuration, time) ANOVA with a post hoc Holm-Sidak procedure for multiple comparisons. Residual volume and drainage efficiency data reported in the table were compared by the Mann-Whitney U Rank Sum test using SigmaStat version 10 (Table 1). P values <0.05 were taken to indicate statistically significant effects of the drainage configurations.

Mean values ± SD, Separate: Conventional Separate Drains; Split: Split Drain Prototype.

## Results

### First series

#### Drainage of D5W

**Figure 3:**
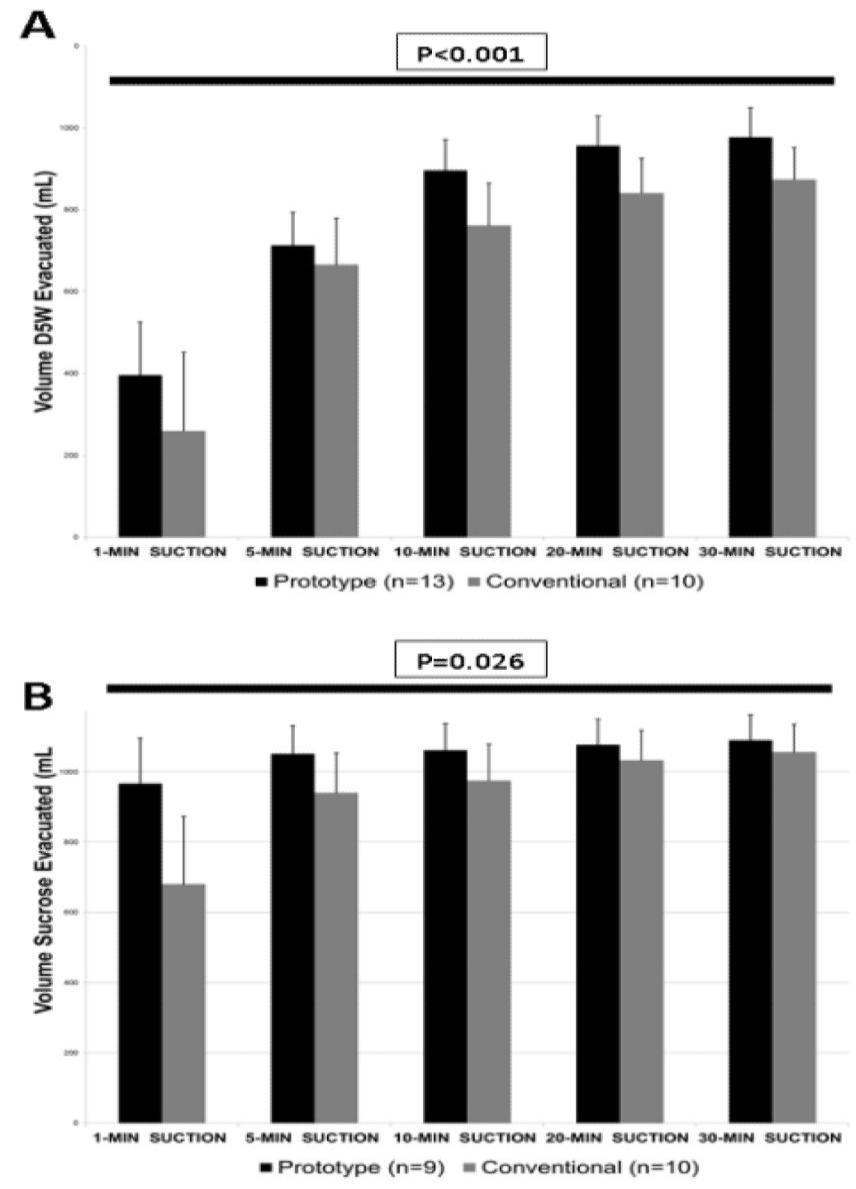
Fluid drainage by conventional separate tubing (gray bars) and split tube prototype (black bars). Fluid drainage was begun immediately after introduction of 1 litre D5W (Panel A) or 0.58 M sucrose (Panel B) into the thoracic cavity, and was monitored for 30 min. Mean values ± SD; numbers of experiments are indicated in the Table. The overall P values for the ANOVA comparing the two configurations are indicated above the horizontal bar in each panel.

The first series of experiments compared drainage of 5% dextrose, i.e. D5W, by the split drain vs. conventional separate drains. At 1 min, the split drain prototype extracted 401 ± 253 ml vs. 279 ± 199 ml by the separate drains (P=0.18; Figure 3A). By 30 min, the split drain prototype had extracted 1029 ± 122 ml vs. 943 ± 202 ml by the conventional configuration (P=0.34; Table). The split drain left a markedly lower residual volume (53 ± 99 mL) than did the conventional separate drains (148 ± 121 ml; P=0.007). Drainage efficiency was 95 ± 10% for the split drain vs. 86 ± 12% for the separate drains (P=0.011). Thus, the split drain removed D5W from the pleural space more efficiently than the standard configuration.

### Second Series

#### Drainage of Hypertonic Sucrose Solution

The results of the first series prompted a second study examining drainage of 0.58 M sucrose, a solution of a viscosity simulating that of plasma. The split drain prototype affected more rapid initial pleural drainage of 0.58 M sucrose than the conventional separate drains (Figure 3B). Within the first 1 min, the split drain prototype extracted 967 ± 129 ml, 42% more than the volume (680 ± 192 ml) extracted by the separate drains (P<0.001). At 5 min, the volume drained by the split tube (1051 ± 80 ml) remained above (P=0.027) that drained by the separate tubes (940 ± 113 ml). Fluid drainage subsequently slowed in both groups. By 30 min, fluid drainage had essentially ceased in both groups; evacuated volumes (Table) were 1089 ± 72 ml by the split drain and 1056 ± 78 ml by the separate drains (P = 0.5). The split drain tended to leave a lower residual volume (25 ± 10 ml) than the conventional drain (62 ± 23 ml) (P=0.128), and drainage efficiency by the split configuration (98 ± 1%) was at least as high as that of the separate drains (95 ± 6%; P=0.111). Thus, the split tubing evacuated 0.58 M sucrose more rapidly than and at least as effectively as the standard drain.

## Discussion

After thoracic operations, fluid effuses into the thoracic cavity from surgical incisions in the pleural and pericardial membranes, thoracic organs and blood vessels [1-4]. If enough fluid accumulates to compress the heart and/or lungs, clinically significant impairment of cardiac and/or pulmonary function may ensue [2,5-9]. Accordingly, the placement of chest tubes to affect thoracic drainage is the standard of care for management of postoperative thoracic effusions. These tubes are exteriorized through incisions in the chest wall, which raises the risk of infection, can produce discomfort, and are prone to displacement, especially when the patient resumes ambulation.

The limitations of conventional chest drains prompted development and evaluation of a novel split drainage system, with a Y-shaped tubing configuration permitting separate placement of two openings within the thorax. The two arms of the drain converge into a common tube exteriorized through a single chest incision. An external wire serves to impart deformability to the tubing to help ensure the tubes hold their shapes and positions after installation in the chest.

The performance of the novel split drain system proved to be superior to conventional chest drainage with separate tubes by some measures. Compared with conventional thoracic drainage, the novel system affected a higher rate of evacuation of fluids of similar viscosity to that of post-surgical effusions. These pre-clinical results indicate that precise positioning of separate arms of the novel chest drain in the costo-diaphragmatic recess and mediastinum is as efficient and affords more complete fluid evacuation than conventional, completely separate drains. A higher rate of fluid evacuation could reduce indwelling time and improve patient comfort and enhanced thoracic drainage could ameliorate some of the most prevalent post-surgical complications associated with cardiac surgery [5]. These results also underscore the importance of proper placement of chest drains to ensure efficient post-surgical thoracic drainage.

## Limitations

It is acknowledged that the split drain was evaluated under controlled conditions in an anesthetized, recumbent experimental animal, not in a potentially ambulatory, awake patient. The fluid was introduced into the thoracic cavity via a catheter, and did not originate from thoracic effusions. Drainage was monitored for 30 min, at which time the split drain had evacuated a volume in excess of the amount introduced. In the clinical setting, chest drains are kept in place for days following cardiothoracic surgery [4,9]. Additionally, it is possible that a blockage originating from either arm of the split drain may obstruct fluid evacuation via the common outlet to a reservoir. However, since both the split and conventional configurations connect to the reservoir via a Y-adaptor, both are susceptible to obstructions at the Y-connection. Thus, additional testing and refinements are necessary before the split drain can be introduced to clinical practice.

## Future Refinements

This study evaluated a first-generation split drain prototype. This prototype was fitted with an external wire coil and wrapped with a biocompatible film, permitting accurate bending and positioning of the drainage tubes in the desired locations within the thoracic cavity, and maintained the tubing configuration so it stayed in position throughout the experiment. Such an external wire would be unsuitable for clinical application. Instead, the split drain must be refined to contain embedded, malleable, biocompatible wires, permitting it to be surgically implanted, precisely positioned to optimize drainage, and withdrawn with minimal trauma to the pleural membranes and chest wall. In addition, it will be desirable to develop smaller-caliber tubing to minimize discomfort and facilitate earlier post-surgical ambulation. Indeed, small-caliber tubing has been found suitable for chest drainage after cardiopulmonary bypass [9]. Smaller-caliber tubing is more flexible and thus, more susceptible to dislodgement, so the incorporation of malleable components will be essential to ensure small-caliber tubing retains the desired shape and placement in a split-drain configuration.

## Figures and Tables

**Table 1:** Extent and efficiency of thoracic drainage by conventional vs. experimental drainage systems

	D5W			0.58 M Sucrose		
	Separate	Split	P	Separate	Split	P
n	9	12		10	9	
Volume Drained, ml	943 ± 202	1029 ± 122	0.34	1056 ± 78	1089 ± 72	0.5
Residual Volume, ml	148 ± 121	53 ± 99	0.007	62 ± 72	25 ± 10	0.128
Drainage Efficiency, %	86 ± 12	95 ± 10	0.011	95 ± 6	98 ± 1	0.111
